# *Chest In Time*: Investigating the utility of a novel X-ray based blunt chest trauma clinical prediction model in a resource-limited setting in South Africa

**DOI:** 10.4102/sajr.v30i1.3344

**Published:** 2026-03-06

**Authors:** Avika Kalideen, Tanusha Sewchuran

**Affiliations:** 1 Discipline of Radiology, School of Medicine, University of KwaZulu-Natal, Pietermaritzburg, South Africa

**Keywords:** chest trauma score, blunt chest trauma, South Africa, chest X-ray, thoracic injury

## Abstract

**Background:**

Blunt chest trauma (BCT) is a frequent manifestation of traumatic injury, either as isolated thoracic injury or in the setting of polytrauma.

**Objectives:**

Several chest trauma scores (CTSs) exist based on clinical, biochemical and imaging findings to assist in the risk stratification of patients who have sustained blunt chest trauma. These injuries are often not initially clinically evident and a risk stratification tool serves to identify patients at risk for pulmonary compromise and to establish early diagnostic and therapeutic strategies to improve morbidity and mortality.

**Method:**

Patient data were obtained from the Greys Hospital Emergency Department’s triage books. The images for patients within the study sample were scored under the categories of age, number of rib fractures, bilaterality of rib fractures, presence and significance of pulmonary contusions, and pleural-based injury. Patients were classified as either critical or non-critical using final disposition as a surrogate. The seminal CTS was initially calculated, following which a novel South African chest trauma score (SA-CTS) was hypothesised and computed based on the chest X-ray alone for ease of applicability and reproducibility in resource-constrained settings.

**Results:**

A conventional CTS ≥ 5 was clinically significant for a critical outcome. However, with the SA-CTS, a score ≥ 4 was found to be statistically significant. Age was not found to be a significant contributing factor. Pleural-based injuries were found to be contributory factors to a critical outcome.

**Conclusion:**

Clinical prediction models serve in the risk stratification and early identification and pre-emptive management of patients at high risk for clinical decompensation. Based on the results, the incorporation of the SA-CTS into daily practice in the management of BCT patients is proposed.

**Contribution:**

This will prove especially useful in triggering early up-referral for both advanced imaging and tertiary level care in a resource limited setting.

## Introduction

Chest trauma is currently listed as the third most common manifestation of trauma following head and extremity injuries.^1^ As a result of blunt chest trauma (BCT), patients with increasing complexity or injury extent are more likely to develop respiratory sequelae, exacerbating their injury profile and complicating their disease trajectory, which contributes to a high level of morbidity and mortality.^2,3,4^ Furthermore, in BCT, additional patient factors including delayed presentation, age over 50 years, bilaterality of injuries, associated extra-thoracic injury and patients in need of intensive care unit (ICU) admission, negatively affect prognosis.^1,2,3^ Immediate identification of BCT patients at an increased risk of pulmonary compromise is challenging, as the full extent of the insult is often not apparent at initial presentation.^3,5^ This becomes even more complex in the context of effective resource allocation, a daily challenge experienced in low-to-middle income countries (LMICs).^6^

Several clinical prediction models, using imaging and clinical variables to generate an additive score, have been evaluated as risk stratification tools to predict patients at high risk for clinical decompensation.^3,7^ Table 1 highlights some currently known iterations of these clinical prediction models, the imaging modality utilised and the predicted patient outcomes.^7^ High scoring patients should impact a clinician’s decision making, with the ideal objective being to trigger advanced imaging, to take pre-emptive resuscitative methods, provide early ventilation and early referral to a more specialised, closely monitored environment.^7^ These environments, like high care facilities or ICUs, as well as access to advanced imaging facilities, are a limited and over-burdened resource in government health institutions in South Africa.^8,9^

**TABLE 1:** Tabulated comparison between some main blunt chest trauma clinical prediction models.

Score	Author	Data requirements	Components	Outcome assessed
TTSS	Pape et al.^14^	CXR CT Biochemical	Age Pulmonary contusion Pleural-based involvement Rib fractures PF ratio	In-hospital mortality Pulmonary complications Respiratory failure ICU factors including length of stay
CTS	Pressley et al.^10^	CXR CT	Age Rib fractures Bilaterality of rib fractures Pulmonary contusion	Mortality Pulmonary compromise Pulmonary complications Mechanical ventilation
Rib Score	Chapman et al.^15^	CT	Rib fractures Bilaterality of rib fractures Displaced rib fractures First rib fractures Flail chest	Pulmonary complications
Battle Score	Battle et al.^3,7,16^	CT	Age Number of rib fractures Bilaterality Pulmonary contusions Thoracic haematomas Entrance to ICU	Analgesia requirements ICU admission Medical emergency team calls
STUMBL	Battle et al.^3^ 2014 Callisto et al.^17^	CT Biochemical	Age Number of rib fractures Use of anti-coagulation Comorbid pulmonary disease Recorded SpO2	Analgesia requirements ICU admission

Note: Please see full reference list of Kalideen A, Sewchuran T. *Chest In Time*: Investigating the utility of a novel X-ray based blunt chest trauma clinical prediction model in a resource-limited setting in South Africa. S Afr J Rad. 2026;30(1), a3344. https://doi.org/10.4102/sajr.v30i1.3344, for more information.

TTSS, thoracic trauma severity score; CTS, chest trauma score; STUMBL, STUdy of the Management of BLunt chest wall trauma; SpO2, saturation of peripheral capillary oxygen; PF ratio, PaO2/FiO2, arterial oxygen partial pressure/fractional inspired oxygen.

One of the proposed prediction models, the conventional chest trauma score (C-CTS) was first described by Pressley et al.^10^ in 2012 in a level 1 state-designated trauma centre in the United States of America.^10^ This clinical prediction model represents a simple scoring system used to risk stratify patients based on the CXR or CT, utilising certain patient demographic and imaging factors in patients who have sustained BCT. These factors include age, number of rib fractures, bilaterality of rib fractures and the presence of pulmonary contusions. The C-CTS is an additive score with a minimum score of 2 and a maximum score of 12, using the algorithm described in Table 2. A score of ≥ 5 was described as being significant for the development of pulmonary compromise.^10^

**TABLE 2:** Score attributed to each variable required for the conventional chest trauma score.

Score	Age of patient (years)	Number of rib fractures	Bilateral rib fractures	Pulmonary contusion
0	-	-	No	None
1	< 45	< 3	-	Unilateral minor
2	45–65	3–5	Yes	Bilateral minor
3	> 65	> 5	-	Unilateral major
4	-	-	-	Bilateral major

*Source:* Pressley CM, Fry WR, Philp AS, Berry SD, Smith RS. Predicting outcome of patients with chest wall injury. Am J Surg. 2012;204(6):910–913; discussion 3–4. https://doi.org/10.1016/j.amjsurg.2012.05.015

Note: Conventional chest trauma score calculation = Age score + Rib fracture score + Bilateral rib fracture score + Pulmonary contusion score.

The C-CTS was further validated by Chen et al.,^11^ also at a level 1 trauma centre in the United States, who established that using a C-CTS ≥ 5 corresponded to at least a fourfold increase in patient mortality.^11^ Subsequently Harde et al.^12^ and Hussein et al.^13^ assessed the same score in India and Iraq, respectively, where the socioeconomic circumstances more closely align with those of South Africa. These authors found scores of 5 (Harde et al.^12^) and 5.5 (Hussein et al.^13^), respectively, which were significantly associated with poor clinical outcomes.^12,13^

Drawing on the data presented by Chen et al.,^11^ Harde et al.^12^ and Hussein et al.,^13^ this study aimed to assess the viability of the C-CTS in a resource-limited South African setting. Furthermore, based on the analysis of the C-CTS within the current study sample, a novel South African CTS (SA-CTS) is proposed as a surrogate for predicting the clinical trajectory in a resource-limited setting based on the CXR alone. Both validation of an existing score and the proposal of a novel score have not been performed in a South African or sub-Saharan African context to the best of the authors’ knowledge. This underscores the rationale for this study in a resource-constrained setting with a significantly heavy trauma burden.

## Research methods and design

### Data collection

Patients were retrospectively identified from written hospital triage records in the emergency department (ED) over a period of 3 years (2022 to 2024). Inclusion criteria were patients ≥ 18 years of age, who presented or were referred to Greys Hospital with a mechanism of injury in keeping with, but not isolated to, BCT and where the patient’s baseline CXR was available on the in-house electronic Picture Archiving and Communication System (PACS) and Radiology Information System (RIS).

Demographic information (including biological sex, year of injury, year of birth, hospital number, mechanism of injury, mechanical ventilatory status in the ED and initial disposition) was retrieved from the triage book. For the purpose of this study, the disposition was defined as either: (1) discharged (the patient did not require further medical treatment), (2) ward (the patient was referred to a general ward post initial examination either at Greys Hospital or transferred back to their base referral hospital), (3) ICU (the patient was referred to a highly monitored ward post initial examination), (4) poor outcome (the patient was T-pieced for the purpose of palliative oxygenation) or (5) death (patient demised in the ED prior to a subsequent disposition).

### Radiographic assessment

Digitised CXRs were evaluated by an independent specialist radiologist, who was blinded to the reports and patient outcomes. The following imaging-based criteria were assessed: the number of rib fractures, the presence of bilateral rib fractures, the presence and extent of pulmonary contusion, the presence of pneumothorax and the presence of haemothorax. Pulmonary contusion was classified as unilateral or bilateral and further categorised as minor (< 50% lung field opacity) or major (> 50% of lung field opacity). Widened mediastinum, usually of concern in the context of BCT, was excluded because the majority of X-rays were acquired supine in an acute setting, limiting diagnostic accuracy.

### Assessment of a South African CTS

In South Africa, the availability of imaging modalities remains significantly varied between institutions, with cross-sectional imaging often being available only at tertiary or referral centres.^8^ Therefore, to have the greatest applicability in South Africa and other LMICs, a comprehensive scoring system would need to use CXR as the primary modality for assessment of the extent of trauma. Taking this into consideration, the C-CTS was modified to re-categorise the score assigned to the absence of rib fractures, exclude age based on various factors and include two additional pleural-based injuries, namely pneumothorax and haemothorax.

The first revision was made to the rib scoring allocations from the C-CTS to the SA-CTS. The SA-CTS proposed the addition of a further criterion for the absence of any visible rib fractures. While the previous scoring system assigned a score of one to the presence of less than three ribs fractures, a score of zero was assigned to the absence of any visualised rib fractures on CXR to clearly distinguish the presence of injury from the absence of injury, as this impacts the ultimate score and further management.

The second modification was the removal of the age criteria, the reason for which was multifactorial. The trauma burden in LMICs tends to impact a predominantly younger population where a high violent trauma burden is often linked to youth unemployment and poverty.^8^ This is mirrored in the study sample as the majority of the dataset was under the age of 45. This yields a relatively skewed study sample in terms of the age demographic creating an unintentional bias with respect to applying traditional scoring systems.

With respect to the addition of pleural-based injury variables, this was originally included as part of the TTTS, a different clinical prediction model by Pape et al.^14^ Given the common occurrence in a South African setting of pleural-based injuries and the high likelihood of these injuries occurring in conjunction with rib fractures because of proximity, the presence of pleural-based injuries was included in the SA-CTS.^18,19^ The final SA-CTS can therefore range from a minimum of 0 to a maximum of 11. Scores attributed to each variable are provided in Table 3.

**TABLE 3:** Score attributed to each variable required for the novel South African chest trauma score.

Score	Number of rib fractures	Bilateral rib fractures	Pulmonary contusion	Pneumothorax	Haemothorax
0	0	No	None	No	No
1	1–2	-	Unilateral minor	Yes	Yes
2	3–5	Yes	Bilateral minor	-	-
3	> 5	-	Unilateral major	-	-
4	-	-	Bilateral major	-	-

Note: Proposed South African chest trauma score calculation = Rib fracture score + Bilateral rib fracture score + Pulmonary contusion score + Pneumothorax score + Haemothorax score.

### Statistics

Data were documented and manually curated in Microsoft® Excel (version 16.93). Descriptive statistics were also conducted in Microsoft® Excel. Prior to the assessment of C-CTS and SA-CTS in this study sample, the association between each variable under study and patient outcome was assessed using the Fisher’s exact test in the R foundation for Statistical Computing platform (R version 4.2.2, The R foundation for Statistical Computing, Vienna, Austria). A *p*-value < 0.05 was considered statistically significant for all association tests.

### Ethical considerations

This retrospective study was undertaken at Greys Hospital, a tertiary healthcare facility in Pietermaritzburg, KwaZulu-Natal (KZN), South Africa. Gatekeeper permission was granted by the hospital on 20 January 2025. The study was then approved by the KZN Department of Health on 31 January 2025 (reference number: KZ_202501_019). The Biomedical Research Ethics Committee (BREC) of the University of KwaZulu-Natal granted final ethical approval (reference number: BREC/00008110/2024) on 06 February 2025. Data retrieved during this study were anonymised.

## Results

Clinical information and baseline CXRs from a total of 315 adult patients 18 years or older, were obtained. Fourteen patients were excluded from this study because of missing data, with a final included total of 301 patients.

Table 4 stratifies the mechanism of injury, demographic and radiographic features as well as the disposition of patients within the sample. The vast majority of patients were male (70.8%) and under the age of 45 years (76.1%). On CXR, the majority of patients presented with less than three rib fractures (*n* = 259, 86%), while a minority had bilateral rib fractures (*n* = 14, 4.7%). Lung fields were clear in 108 (35.9%) patients, 114 (37.9%) patients had minor pulmonary contusions, while 79 (26.2%) had major pulmonary contusions. The majority of patients had an absence of pleural-based injuries: 91.4% and 62.5% for the absence of pneumothorax and haemothorax, respectively.

**TABLE 4:** A summary of the study sample (*N* = 301).

Criteria	*n*	%
**History**		
Injury		
Motor vehicle accident	181	60.1
Pedestrian vehicle accident	90	29.9
Fall from height	21	7.0
Assault	6	2.0
Missing mechanism data	3	1.0
**Demographic data**		
Biological sex		
Male	213	70.8
Female	88	29.2
Age (years)		
< 45	229	76.1
45–65	55	18.3
> 65	17	5.6
**Clinical disposition**		
Outcome		
Critical	99	32.8
Death	11	3.7
Poor outcome	2	0.7
ICU	86	28.6
Non-critical	202	67.2
Ward	200	66.4
Discharged	2	0.7
Ventilated		
No	269	89.4
Yes	32	10.6
**Radiographic features**		
Number of rib fractures		
0	190	63.1
1–2	69	23.0
3–5	41	13.6
> 5	1	0.3
Bilateral rib fractures		
No	287	95.3
Yes	14	4.7
Pulmonary contusion		
None	108	35.9
Unilateral minor	77	25.6
Bilateral minor	37	12.3
Unilateral major	53	17.6
Bilateral major	26	8.6
Pneumothorax		
No	275	91.4
Yes	26	8.6
Haemothorax		
No	188	62.5
Yes	113	37.5

ICU, intensive care unit.

**FIGURE 1:**
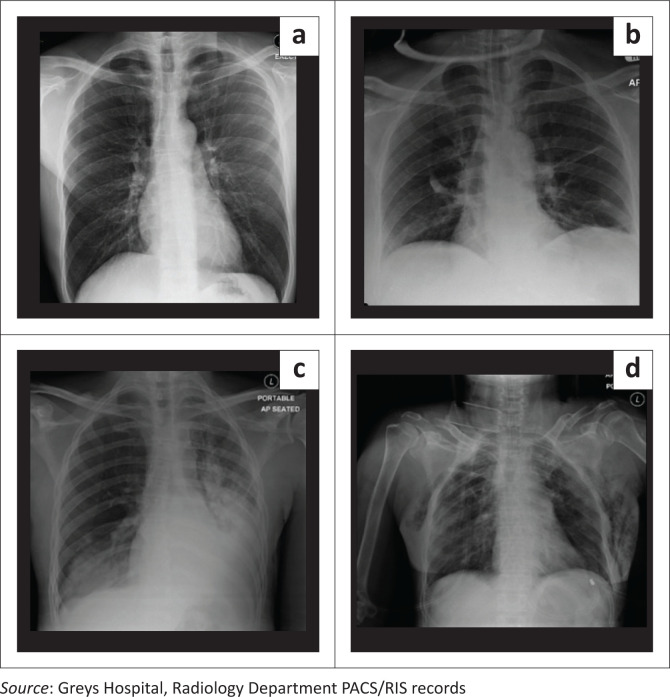
Images of blunt chest trauma patients depicting various chest trauma scores. (a) A 27-year-old man with a history of fall from height; no rib fractures, pulmonary contusions or pleural-based injury; C-CTS 2 and SA-CTS 0. (b) A 53-year-old man involved in a motor vehicle accident (MVA) depicting unilateral right-sided minor pulmonary contusions; no associated pneumothorax or haemothorax; C-CTS 3 and SA-CTS 1. (c) A 31-year-old man post-MVA and subsequently transferred to the ward, with bilateral major pulmonary contusions and left-sided haemothorax; C-CTS 5 and SA-CTS 5. (d) A 56-year-old man involved in a pedestrian vehicle accident who was subsequently intubated, ventilated and transferred to ICU, with multiple bilateral rib fractures, bilateral major pulmonary contusions and pneumothoraces; C-CTS 11 and SA-CTS 10.

Figure 1 displays an image series of progressively increasing CTS values, some of which demonstrate the presence of rib fractures, pulmonary contusions and pleural-based injuries (labels a–d). The lower scoring C-CTS values, images A and B, in fact, score lower on the SA-CTS than the C-CTS. This strengthens the rationale behind the SA-CTS, which is aimed at the risk stratification between potential critical and non-critical patients based on CXR only. Those with lesser injury extent reflect those patients at a lower risk, thereby limiting potentially unwarranted up-referrals to overburdened resource-limited tertiary centres.

### Association of clinical variables and radiological features with outcome

The Fisher’s exact test was used to determine any associations between clinical variables and outcome as well as radiological features and outcome (Table 5). Statistically significant associations were observed between the following clinical variables and outcome: number of rib fractures (*p* < 0.001), the extent of pulmonary contusion (*p* < 0.001), pneumothorax (*p* < 0.001) and haemothorax (*p* = 0.003).

**TABLE 5:** Association testing results between variables and outcome.

Variable	Outcome		*p*	95% CI	Odds ratio
	Critical	Non-critical			
Biological sex	-	-	0.500	0.45–1.42	0.80
Male	73	140	-	-	-
Female	26	62	-	-	-
Ventilation	-	-	< 0.001* (8.71 × 10^−13^)	8.04–143.68	27.16
Not ventilated	70	199	-	-	-
Ventilated	29	3	-	-	-
Age (years)	-	-	0.060	-	-
< 45	81	148	-	-	-
45–65	11	44	-	-	-
> 65	7	10	-	-	-
Number of rib fractures	-	-	< 0.001* (6.96 × 10^−4^)	-	-
< 3	75	184	-	-	-
3–5	23	18	-	-	-
> 5	1	0	-	-	-
Presence of bilateral rib fractures	-	-	0.020*	1.14–15.33	3.92
No	90	197	-	-	-
Yes	9	5	-	-	-
Pulmonary contusion	-	-	< 0.001* (1.66 × 10^−7^)	-	-
None	20	88	-	-	-
Minor unilateral	20	57	-	-	-
Minor bilateral	12	25	-	-	-
Major unilateral	30	23	-	-	-
Major bilateral	17	9	-	-	-
Pneumothorax	-	-	< 0.001* (9.77 × 10^−5^)	2.11−14.84	5.35
No	81	194	-	-	-
Yes	18	8	-	-	-
Haemothorax	-	-	< 0.001* (3.51 × 10^−3^)	1.25–3.56	2.11
No	50	138	-	-	-
Yes	49	64	-	-	-

CI, confidence interval.

A statistically significant association was observed between ventilatory support and outcome (*p* < 0.001), where 91% of ventilated patients (*n* = 29 patients) were either transferred to a high or intensive care setting or demised. Based on odds ratio, ventilated patients were 27.13 times more likely to have a critical outcome. The remainder of these patients were discharged post T-piece for palliative oxygenation. This result, however, should be treated with caution because of a wide confidence interval (CI), likely attributable to the small sample size.

### Calculation of conventional chest trauma scores

**FIGURE 2:**
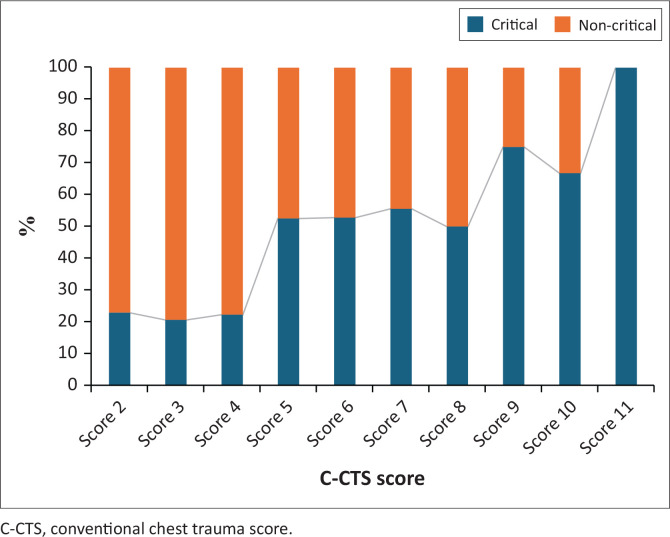
Conventional chest trauma scores with patient outcomes.

As displayed in Figure 2, based on the C-CTS, the percentage of patients with a critical outcome increased sharply from a score of 5. A maximum score of 12 was not observed in this study.

### Calculation of South African chest trauma score

**FIGURE 3:**
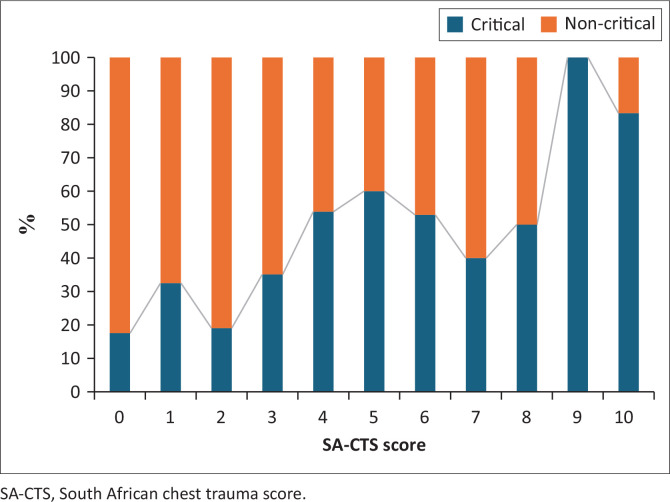
Proposed South African chest trauma score with patient outcomes.

As displayed in Figure 3, the percentage of critical patients increased from a score of 4 when applying the SA-CTS. A maximum score of 11 was not observed in this study.

The relevant scores for the C-CTS and SA-CTS could be consolidated into two categories: low (includes C-CTS of 2, 3 and 4 and SA-CTS 1, 2, 3) and high (C-CTS of 5 and above and SA-CTS of 4 and above) as demonstrated in Table 6. A significant associated between high C-CTS and critical outcome was observed where based on odds ratio, patients with a C-CTS of 5 or higher were at a 4.34 increased risk of a critical outcome. A significant association between high SA-CTS and critical outcome was also observed where based on odds ratio patients with a SA-CTS of greater than or equal to 4 were at a 4.17 increased risk of a critical outcome.

**TABLE 6:** Results of association testing between conventional chest trauma score and South African chest trauma score categories and outcomes.

Score	Total	Outcome		*p*	95% CI	OR
		Critical	Non-critical			
**C-CTS**	-	-	-	< 0.001 (2.514 × 10^−8^)	2.52−7.55	4.34
Low (≤ 4)	201	44	157	-	-	-
High (≥ 5)	100	55	45	-	-	-
**SA-CTS**	-	-	-	< 0.001 (1.275 × 10^−7^)	2.38−7.90	4.17
Low (≤ 3)	216	51	165	-	-	-
High (≥ 4)	85	48	37	-	-	-

CI, confidence interval; OR, odds ratio; C-CTS, conventional chest trauma score; SA-CTS, South African chest trauma score.

## Discussion

The vast majority of trauma deaths occur in LMICs.^8^ South Africa is no exception because of a high prevalence of violence and injury, with trauma representing one of the pillars of the quadruple burdens of disease, the characteristics of which are primarily violent, having a young male predominance.^8,9,17^ Coupled with this, the South African healthcare system is riddled with resource constraints, some of which of relevance to this study include, limited access to healthcare facilities and services, and access to cross-sectional imaging. The study setting, Greys Hospital, which is located in the uMgungundlovu district, is a tertiary facility with only one CT scanner offering 100% of tertiary level services to the uMgungundlovu, Uthukela, UMzinnyathi, Amajuba and Harry Gwala districts occupying western KZN, which according to the last 2022/2023 annual report, serves a population of approximately 4 million people.^20^

Pre-emptive optimisation of pre-hospital and hospital care, including appropriate up-referral of patients with timeous escalation to prevent the development of complications, is the goal to improve clinical outcomes and limit patient morbidity and mortality.^8^ This task is challenging both clinically and logistically with respect to effective resource allocation.^3,4,8,21^ Therefore, to assist in the identification of high-risk patients, several image-based clinical prediction models have become the focus of study in many countries.^7^ An ideal and efficacious CTS, particularly in a LMIC, requires the use of a universally accepted, commonly available resource and must represent a score that is easily calculable by clinicians working in both rural and urban settings alike. This necessitates the use of the CXR in LMICs, despite CT being the modality of choice in most recent clinical prediction models for the assessment of BCT patients.^7,8,22^

The incorporation of a novel SA-CTS to assist with risk stratification and basic prognostication based on the CXR in a resource-constrained setting is proposed. In concordance with previous studies, retrospective evaluation of the C-CTS in a South African study sample demonstrated that a C-CTS of ≥ 5 is significantly associated with critical patient outcomes. However, relevantly, the SA-CTS demonstrated that a score of ≥ 4 had a statistically significant association with critical patient outcomes.

A few adaptations to the C-CTS from the seminal study were made to construct the SA-CTS.^10^ Firstly, the rationale for the recategorisation of the rib fracture scoring was to differentiate between the presence and absence of injury as this impacts the final score and subsequently immediate management. Secondly, the age criterion was removed because of the inherently skewed dataset where the majority of the patients were under the age of 45 years, a statistic which is representative of the trauma burden of disease in South Africa.^8,9^ When an additional modified SA-CTS was performed (representing the SA-CTS with the inclusion of age, named the modified SA-CTS) the result was the same as the C-CTS where a score of ≥ 5 was the fulcrum between critical and non-critical outcomes (Online Appendix 1). The removal of age alone changed the critical point of the score to ≥ 4. While the significance of the proposed removal of age from the SA-CTS needs to be confirmed in a larger dataset, age remains a consideration when holistically assessing a patient in a resource-constrained setting.^23^ Finally, the rationale behind the addition of pleural-based injuries was because of the common occurrence of these injuries in clinical practice and the probability of these occurring in conjunction with rib fractures, a factor that is already a variable within the score.^18,19^ Both pneumothorax and haemothorax were directly associated with outcome in this study.

Notably, the study sample revealed outliers in establishing the novel SA-CTS. The primary theory for these discordant phenotypes is thought to be because of the presence of multisystemic injuries in polytrauma patients, where BCT rarely occurs in isolation. When analysing low scoring patients with critical outcomes, it was found that many sustained significant head or intra-abdominal trauma while their thoracic cage was relatively spared.

In contrast, with respect to high scoring patients with non-critical outcomes, the demographics of these patients were consistently older than 45 years, a demographic that is relatively under-represented in this study sample. A potential explanation for the relative higher scores of older patients is that the interpretation of baseline CXR may have been obscured because of the presence of concomitant pre-existing comorbidities and longstanding pulmonary pathology findings, which may be attributed to trauma in the acute setting. Interestingly, when analysing higher scoring older patients, these patients were predominantly admitted to a general ward, emphasising the resource-constrained climate, while underestimating their relative clinical stability in the setting of a potentially biased CXR score, a notion that is confirmed in published literature.^24^ In an ideal setting, this patient population should have been monitored in a high care environment, based on age alone because of higher risk of clinical decompensation.^23,24^

**FIGURE 4:**
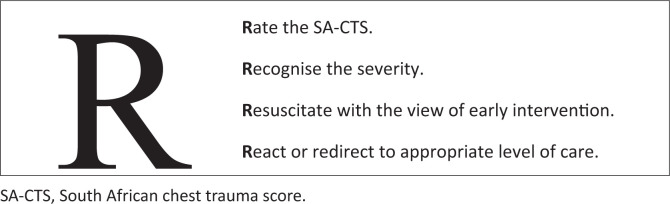
The proposed four “Rs” for routine rapid assessment of polytrauma patients in the context of BCT.

Upon validation of these findings in a larger study sample, the use of the SA-CTS as a clinical prediction model to improve morbidity and mortality in patients with BCT with the addition of the four Rs (Figure 4) into routine rapid assessment is proposed: (1) *Rate* the score of the CXR using the SA-CTS, (2) *Recognise* the severity, (3) *Resuscitate* pre-emptively, as early intervention improves outcome, (4) *React or re-direct* the patient to a higher level of care for assessment, further imaging or for admission into a highly monitored area.

Study limitations include the small sample size. The study site was a tertiary hospital where the majority of patients were referrals from district or regional base hospitals. As such, the extremes of presentation because of BCT may be missed as a result of their non-referral. Patients who presented with a hard copy CXR film from base were not captured on the Greys Hospital electronic system, and therefore excluded. Furthermore, specific demographic data were, at times, unavailable because of poor note-keeping. In addition, the mechanism of injury was used as a surrogate for the presence of BCT, creating some bias in patients with multiple injuries, affecting disposition and outcome.

Although this study aimed to validate the use of the CXR in prognosticating patients, it is widely recognised that CT is the diagnostic imaging modality of choice with respect to patients with BCT. The use of the CXR alone is not without challenges. Firstly, a larger degree of interobserver variability exists especially in the presence of more nuanced injuries. Secondly, the presence of less obvious injuries, such as apical pneumothoraces or subtle pulmonary contusions are often more elusive on CXR than on CT, even to the experienced eye, resulting in overall underscoring in any clinical prediction model, thus underestimating the extent of injury. However, with the local burden of trauma, the practical availability of CT scans for all patients based solely on the mechanism of injury is not feasible. Therefore, it is proposed that the CXR remains the modality of choice for risk stratification as part of a clinical prediction model in a resource-limited setting, while overall, CT remains the diagnostic modality of choice.

## Conclusion

The integration of the CXR-based SA-CTS into daily practice in the assessment and risk stratification of multisystemic polytrauma and isolated BCT patients is proposed. This score aims to pre-emptively streamline patient referral pathways, particularly in younger patients, with the criteria for up-referral being to facilitate further imaging, trigger early intervention strategies or for escalation of care to a highly monitored area. Given the high trauma burden and strained tertiary hospital facilities this could significantly assist with resource allocation.
